# Incidentally Discovered Mucosal Prolapse of the Colon During Colorectal Cancer Screening: A Case Report

**DOI:** 10.7759/cureus.37958

**Published:** 2023-04-21

**Authors:** Mesay H Asfaw, Sneha Adidam, Farshad Aduli, Angesom Kibreab, Joseph Asemota

**Affiliations:** 1 Internal Medicine, Howard University Hospital, Washington, USA; 2 Gastroenterology, Howard University Hospital/Howard University College of Medicine, Washington, USA; 3 Internal Medicine, Howard University College of Medicine, Washington, USA

**Keywords:** benign colon polyp, screening colonoscopy, colonic neoplasm, colonoscopy and polypectomy, mucosal prolapse syndrome

## Abstract

Colonic mucosal prolapse syndrome is a rare type of non-neoplastic non-inflammatory colorectal polyps that can mimic neoplastic lesions. We present a case of a 65-year-old man with mucosal prolapse syndrome, incidentally, discovered during colorectal cancer screening. The patient was asymptomatic, and his physical exam and laboratory test results were unremarkable. During a colonoscopy, the physician removed three small tubular adenomas and two pedunculated polyps suspicious of neoplasms. Retroflexion revealed small internal hemorrhoids. The histology of the larger polyps revealed mucosal prolapse features, while the smaller polyps displayed features consistent with tubular adenomas. Management involves the removal of associated polyps during colonoscopy, followed by surveillance colonoscopy to detect any recurrent polyps or early signs of colorectal cancer. Accurate diagnosis is crucial to avoid unnecessary interventions and ensure appropriate management.

## Introduction

Colonic mucosal prolapse syndrome is a rare type of non-neoplastic non-inflammatory colorectal polyps, which includes solitary rectal ulcers, hamartomas, inflammatory polyps, inflammatory cloacogenic polyps, inflammatory myoglandular polyps, and proctitis cystica profunda [1]. The term was first introduced in 1983 by du Boulay et al. and describes the histological changes that occur in the mucosa of the colon due to mucosal prolapse [2]. These conditions share common microscopic features such as diamond-shaped crypts, fibromuscular obliteration of the lamina propria, thickening of the muscularis mucosa, and the presence of mixed inflammatory cells [3,4]. As it often mimics neoplastic lesions on endoscopy, it is frequently misdiagnosed. In this report, we present a case of an asymptomatic patient with mucosal prolapse, which was discovered incidentally during a screening colonoscopy and had a gross appearance suspicious of neoplasm.

## Case presentation

**Figure 1:**
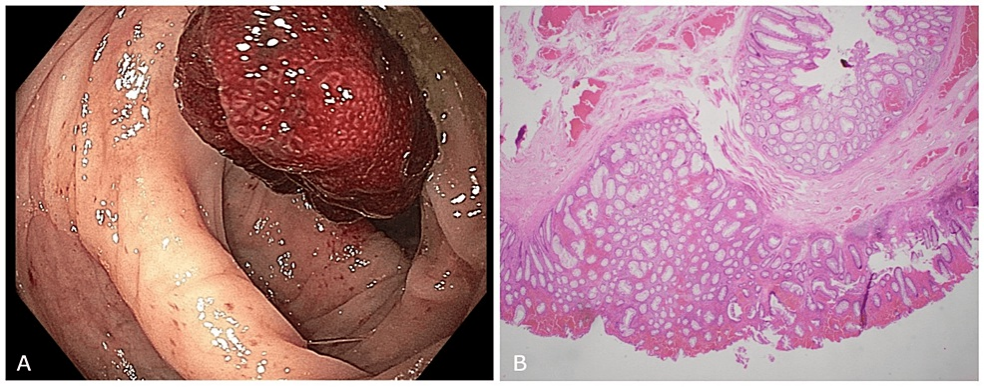
Image taken during colonoscopy (A) and low power microscopy of the polyp (B) done with hematoxylin-eosin stain

A 65-year-old man with a medical history of chronic obstructive pulmonary disease, essential hypertension, and cocaine abuse visited the gastroenterology clinic for a routine colorectal cancer screening. His physical examination revealed no concerning findings, and his laboratory test results were unremarkable, including a normal complete blood count and serum chemistry. During the colonoscopy, the physician removed three small (2mm) polyps with cold forceps from the transverse and descending colon, which were later confirmed to be tubular adenomas. Additionally, two pedunculated polyps, measuring 15mm and 20mm and exhibiting hyperemic features, were detected in the sigmoid and completely removed using a hot snare (Figure 1A). Two clips were placed at the site of the larger polyp removal to minimize the risk of post-polypectomy complications. There was no evidence of diverticulosis and retroflexion revealed small, grade I, non-bleeding internal hemorrhoids. The histology of the 15mm and 20mm polyps revealed colonic mucosa with hyperplastic changes and mucosal prolapse features, while the 5mm and 6mm polyps displayed features consistent with tubular adenomas (Figure 1B). After confirmation of the benign characteristics of the polyps, the patient was scheduled for a surveillance colonoscopy per guidelines.

## Discussion

Colonic mucosal prolapse is often misdiagnosed due to its varied clinical presentation, which can mimic other pathologic conditions. As a result, the true prevalence of this condition is largely unknown, as it is often not recognized or misdiagnosed [5]. Mucosal prolapse typically affects people in their fourth to sixth decade of life and is more common in males than females (3:1 ratio) [6]. Symptoms may include chronic constipation with straining, recurring abdominal pain, altered bowel habits, tenesmus (the sensation of incomplete emptying), partial incontinence, and gastrointestinal bleeding (either occult or gross). The exact mechanism causing mucosal prolapse is not fully understood, but it is believed that spastic contraction of the bowel wall during defecation in patients with chronic straining leads to mucosal redundancy, passive venous congestion, and obstruction [5,6]. Colonic mucosal prolapse can be a manifestation of more serious conditions, such as inflammatory bowel disease and colorectal cancer. Therefore, it is crucial to thoroughly evaluate patients with colonic mucosal prolapse to rule out these underlying conditions.

Endoscopically, mucosal prolapse can be classified into three categories based on its macroscopic appearance: ulcerated lesions (55%), flat erythematous lesions (21%), and polypoid lesions (24%) [7]. Solitary rectal ulcer syndrome (SRUS) is a type of ulcerated lesion that may present with multiple rectal ulcers in up to 22% of cases [8]. The polypoid lesions can be sessile, pedunculated, or broad-based, and include various types of polyps such as inflammatory cap polyps, inflammatory cloacogenic polyps, myoglandular polyps, and mucosal prolapse polyps [9]. While there is no pathognomonic endoscopic finding for mucosal prolapse, the annual tree sign, which is characterized by concentric circular innominate grooves surrounding the lesion seen with chromoendoscopy, has been described as a specific feature that differentiates prolapsed polyps from malignancy [10]. Endoscopic ultrasonography may be helpful in establishing the diagnosis if a prolapsed mucosal fold is suspected to have subepithelial origin [11]. Additionally, certain histologic features such as hyperplastic and serrated changes, crypt branching, hypermucinous epithelial appearance, and regenerative hyperplastic changes like increased mucous cell proliferation and glandular dilatation are characteristic of mucosal prolapse. However, distinguishing mucosal prolapse from entities like villous adenoma can be challenging when villiform epithelial hyperplasia and regenerative atypia are present [12]. Misplaced glands with submucosal mucus lakes can also mimic invasive adenocarcinoma, further complicating the diagnostic process [13].

In this case, the colonoscopy revealed the presence of two pedunculated polyps in the sigmoid colon that exhibited hyperemic features and were completely removed using a hot snare. The histology of these polyps revealed colonic mucosa with hyperplastic changes and mucosal prolapse features. The management of colonic mucosal prolapse involves the removal of any associated polyps during colonoscopy. In this case, the polyps were successfully removed using a hot snare. After the removal of the polyps, a surveillance colonoscopy is recommended to detect any recurrent polyps or early signs of colorectal cancer. The interval between surveillance colonoscopies depends on the number, size, and histology of the polyps removed during the initial colonoscopy. In general, patients with a history of hyperplastic polyps and mucosal prolapse features are at an increased risk of developing recurrent polyps and should undergo surveillance colonoscopy more frequently. It is also important to note that colonic mucosal prolapse can be a manifestation of more serious conditions, such as inflammatory bowel disease and colorectal cancer. Therefore, it is crucial to thoroughly evaluate patients with colonic mucosal prolapse to rule out these underlying conditions [11]. In this case, the patient had a routine colorectal cancer screening, and his physical examination and laboratory test results were unremarkable, suggesting a low suspicion of underlying inflammatory bowel disease or colorectal cancer. In summary, the management of colonic mucosal prolapse, in this case, involved the successful removal of the associated polyps using a hot snare during colonoscopy. A surveillance colonoscopy was also scheduled to detect any recurrent polyps or early signs of colorectal cancer.

## Conclusions

In conclusion, colonic mucosal prolapse is a condition that is often misdiagnosed and has a varied clinical presentation. It is crucial to thoroughly evaluate patients with colonic mucosal prolapse to rule out underlying conditions like inflammatory bowel disease and colorectal cancer. Management involves the removal of associated polyps during colonoscopy, followed by surveillance colonoscopy to detect any recurrent polyps or early signs of colorectal cancer.
